# An Unprecedented Case of p190 *BCR-ABL* Chronic Myeloid Leukemia Diagnosed during Treatment for Multiple Myeloma: A Case Report and Review of the Literature

**DOI:** 10.1155/2018/7863943

**Published:** 2018-10-10

**Authors:** Kosuke Miki, Naoshi Obara, Kenichi Makishima, Tatsuhiro Sakamoto, Manabu Kusakabe, Takayasu Kato, Naoki Kurita, Hidekazu Nishikii, Yasuhisa Yokoyama, Mamiko Sakata-Yanagimoto, Yuichi Hasegawa, Shigeru Chiba

**Affiliations:** ^1^School of Medicine, University of Tsukuba, Tsukuba, Japan; ^2^Department of Hematology, Faculty of Medicine, University of Tsukuba, Tsukuba, Japan

## Abstract

We report the case of a 76-year-old man who was diagnosed as having chronic myeloid leukemia (CML) with p190 *BCR-ABL* while receiving treatment for symptomatic multiple myeloma (MM). The diagnosis of MM was based on the presence of serum M-protein, abnormal plasma cells in the bone marrow, and lytic bone lesions. The patient achieved a partial response to lenalidomide and dexamethasone treatment. However, 2 years after the diagnosis of MM, the patient developed leukocytosis with granulocytosis, anemia, and thrombocytopenia. Bone marrow examination revealed Philadelphia chromosomes and chimeric p190 *BCR-ABL* mRNA. Fluorescence in situ hybridization also revealed *BCR-ABL*-positive neutrophils in the peripheral blood, which suggested the emergence of CML with p190 *BCR-ABL*. The codevelopment of MM and CML is very rare, and this is the first report describing p190 *BCR-ABL*-type CML coexisting with MM. Moreover, we have reviewed the literature regarding the coexistence of these diseases.

## 1. Introduction

Multiple myeloma (MM) is a lymphoid cancer that is characterized by monoclonal proliferation of malignant plasma cells in the bone marrow, monoclonal protein in the serum, and organ dysfunction [1]. Chronic myeloid leukemia (CML) is a clonal disorder of myeloid origin that is characterized by the Philadelphia chromosome, t(9; 22) (q34; q11), in which the *BCR-ABL* fusion gene is created. Several types of *BCR-ABL* are known, with the p210 and p190 types being the most common, distinguished by the break point in the *BCR* gene. The vast majority of CML cases result from the p210-type of *BCR-ABL*, while the p190-type is rarely found in CML [2]. The coexistence of MM and CML in the same patient is rare, with only 22 reported cases [3–23], all involving p210 *BCR-ABL*. Therefore, we report a case of coexisting MM and CML with p190 *BCR-ABL*. Given the rarity of this case, we also discuss the origins of these hematologic malignancies and review the relevant literature.

## 2. Case Report

**Figure 1:**
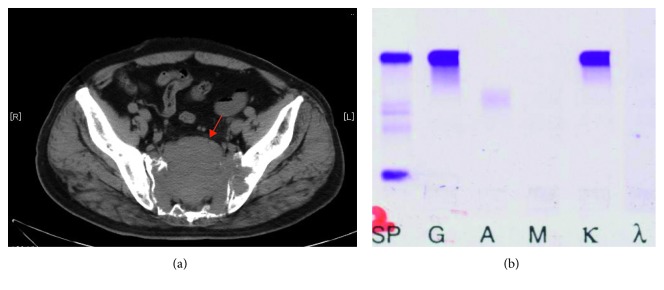
(a) Computed tomography (CT) at the time of multiple myeloma (MM) diagnosis. Sacral tumor and lytic bone involvement in the lumbar spine are shown (arrow). (b) Serum immunofixation at the time of MM diagnosis. SP, size marker; G IgG; A IgA; M IgM; κ, kappa chain; *λ,* lambda chain.

A 76-year-old man was referred to our hospital in September 201X, because of right leg pain, lower back pain, and weight loss of 3 kg. Lumbar magnetic resonance imaging and computed tomography (CT) suggested the presence of lumbar spinal canal stenosis and a sacral tumor (Figure 1(a)). Laboratory testing revealed a markedly elevated serum IgG level (5,436 mg/dL, normal: 800–1,800 mg/dL) and an elevated serum beta-2 microglobulin level (4.1 *µ*g/mL, normal: 0–3 *µ*g/mL), although there were no signs of anemia, renal dysfunction, or proteinuria. Serum immunofixation revealed IgGκ-type M-protein, with an estimated serum-free κ and *λ* chain ratio of 21.5 : 1 (Figure 1(b)). Microscopic examination and flow cytometric analysis of bone marrow aspirate revealed elevated numbers of CD138-positive abnormal plasma cells. Cytogenetic analysis of the bone marrow revealed 46XY, and the patient was diagnosed as having MM (R-ISS, stage II). Chimeric p190 *BCR-ABL* mRNA was not detected in the bone marrow sample at this point. The patient underwent two cycles of bortezomib plus dexamethasone and two cycles of cyclophosphamide, bortezomib, and dexamethasone (CBD) but did not respond to either treatment regimen. The treatment was switched to lenalidomide (25 mg/day) plus dexamethasone (20 mg/week; Ld therapy), and there was a marked response, with a substantial decrease in the M-protein and disappearance of the sacral tumor on CT. After 24 cycles of Ld therapy, the patient achieved a partial response based on the International Myeloma Working Group criteria.

**Figure 2:**
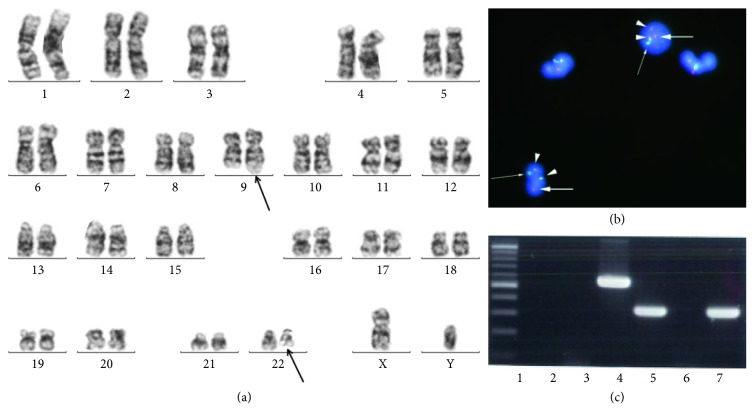
(a) Karyotype from a bone marrow specimen at the time of chronic myeloid leukemia (CML) diagnosis. t(9; 22) is shown by using arrows. (b) Fluorescence in situ hybridization for peripheral blood cells. Arrowheads, *BCR-ABL* fusion gene (yellow); large arrows, *ABL* signal (red); small arrows, *BCR* signal (green). (c) Polymerase chain reaction (PCR) amplification analysis from the bone marrow specimen taken at the time of CML diagnosis. Lane 1, size marker; lane 2, p210 negative control; lane 3, p190 negative control; lane 4, p210 positive control; lane 5, p190 positive control; lane 6, p210 patient's sample; lane 7, p190 patient's sample.

In December 201X+2, the patient developed leukocytosis (white blood cell count: 35.8 × 10^9^/L) and thrombocytopenia (platelet count: 3 × 10^9^/L). Bone marrow biopsy and aspiration revealed hypercellularity with a marked increase in myeloid lineage cells but without an increase in blast cells (4%). Cytogenetic analysis revealed 46XY t(9; 22) (q34; q11.2) in 20 of 20 cells, and fluorescence in situ hybridization (FISH) analysis revealed that 99.5% of the cells were positive for *BCR-ABL*. Peripheral blood neutrophils were also positive for *BCR-ABL* (98.8%) (Figures 2–2). Chimeric p190, but not p210, *BCR-ABL* mRNA was detected by using polymerase chain reaction (Figure 2). The diagnosis was confirmed to be CML with p190 *BCR-ABL* in the accelerated phase (AP), which coexisted with MM (a maintained partial response). Dasatinib treatment (100 mg/day) was started immediately. The dose was subsequently decreased to 50 mg/day, due to the persistence of thrombocytopenia. In April 201X+3, a bone marrow examination indicated that the patient had achieved a second chronic phase, with 31% of this cells being positive for *BCR-ABL* upon FISH analysis, and that his peripheral blood count had normalized. However, 5 months later, FISH analysis revealed that 85.8% of his bone marrow cells were positive for *BCR-ABL*, and subsequently, his treatment was changed from dasatinib to bosutinib. This switch appeared to be ineffective, as no decrease in the *BCR-ABL*-positive bone marrow cells was detected after 2 months.

## 3. Discussion

There have only been 24 cases of coexisting MM and CML reported (Table 1) [3–25]. MM and CML were diagnosed simultaneously in 9 cases, and the diagnoses were sequential in the remaining cases, with MM being diagnosed first in 8 cases and CML being diagnosed first in 7 cases. Among the previous reports, all genotypes confirmed by PCR were p210-CML in patients with coexisting MM and CML. Our case is the first reported of MM coexisting with p190-CML in the same patient. The intervals between the sequential diagnoses ranged from 3 months to 120 months. Multiple possibilities could be considered regarding the association between MM and CML. First, these two malignancies could occur independently, and their coexistence, while very rare, could be simply coincidence. Second, common precursors, referred to as “clonal hematopoiesis of indeterminate potential (CHIP),” give rise to both MM and CML cells in a patient. Given our patient's relatively advanced age, it is possible that CHIP had a common origin, although we have no direct evidence to support this possibility. A third possible explanation is that the second disease might be a therapy-related malignancy that develops after treatment for the first disease. In the present case, the diagnosis of MM was followed by that of CML. The frequency of secondary carcinogenesis in patients with MM was evaluated for cohorts participating in clinical trials with lenalidomide, which were conducted from 2000 through 2012 [24]. This report showed that the incidence of secondary hematologic malignancies was increased in patients who concurrently received melphalan and lenalidomide, while it did not increase in those receiving other treatment protocols, regardless of whether lenalidomide was included [26]. There is no clear evidence that lenalidomide is carcinogenic, and there has only been one reported case of CML that developed after lenalidomide treatment. Therefore, the increased incidence of hematologic malignancies has been attributed to melphalan, because alkylating agents such as melphalan are known to cause therapy-related myelodysplastic syndromes. According to previous case reports of MM coexisting with CML, many patients received alkylating agents as treatment for MM (Table 1). In the present case, a small dose of an alkylating agent (cyclophosphamide; total: 2,400 mg/m^2^) was administered during two cycles of CBD therapy. We, therefore, needed to consider the possibility that the cyclophosphamide treatment caused the CML. However, an accumulation of case reports and further investigations are required to draw definitive conclusions.

Although p190 *BCR-ABL* CML has been reported in 1% of CML cases, there have been several clinical observations suggesting that CML with p190 *BCR-ABL* is difficult to treat [27]. Furthermore, p190 *BCR-ABL* has been identified as a marker for high-risk disease, and early stem cell transplantation has been recommended if the patient is eligible [26]. Our patient received initial treatment of dasatinib for his CML in AP with p190 *BCR-ABL*, which provided a partial cytogenetic response at 4 months after the diagnosis. Although the long-term effects of dasatinib treatment in patients with p190 *BCR-ABL* CML are unknown, in the present case, the duration of response to dasatinib was relatively short.

## Figures and Tables

**Table 1:** Concomitant multiple myeloma and chronic myeloid leukemia cases.

Pt	Age/Sex	Year	First disease	Diagnosis interval (month)	Type of M-protein	Therapy for MM	Therapy for CML	Confirmation method of Ph+	Type of Ph	Reference
1	77M	1972	MM	33	BJP	No treatment	No treatment	Chr		MacSween and Langley [3]
2	65/F	1974	CML	113	IgG-κ	No treatment	Busulfan	Chr		Derghazarian and Whittemore [4]
3	58/M	1982	MM and CML	Simultaneous	IgG-κ	MP, RT	HU, Busulfan,	Chr		Boots and Pegrum [5]
4	71/M	1993	MM	24	IgG-κ	MP, RT	HU	Chr		Klenn et al. [6]
5	72/F	1998	MM and CML	Simultaneous	IgG-κ	VP	IFN-*α*	Chr, PCR, FISH	P210	Tanaka et al. [7]
6	70/M	1999	MM	33	IgG-κ	Not reported	Not reported	Chr		Nitta et al. [8]
7	81/M	2001	MM and CML	Simultaneous	IgA-κ	MP	No treatment	Chr, PCR		Alvarez-Larrán et al. [9]
8	66/M	2003	MM and CML	Simultaneous	IgG-κ	MP	INF-*α*, HU, Busulfan	Chr, PCR, FISH	P210	Schwarzmeier et al. [10]
9	47/M	2003	MM	33	BJP	LOAD-IN	Not reported	Chr		Nakagawa [11]
10	68/M	2005	CML	20	IgG-*λ*	MP	IFN-*α*, imatinib	Chr, PCR	P210	Garipidou et al. [12]
11	85/F	2005	MM and CML	Simultaneous	IgG-*λ*	Not reported	Not reported	Chr, PCR	P210	Wakayama et al. [13]
12	76/M	2009	CML	14	IgA-κ	MP	IFN-*α*, imatinib	Chr, PCR, FISH	P210	Galanopoulos et al. [14]
13	57/F	2009	CML	65	IgA-κ	TD, VAD	Imatinib	Chr, PCR	P210	Michael et al. [15]
14	72/F	2010	CML	3	IgG-κ	No treatment	Imatinib	Chr, PCR	P210	Ide et al. [16]
15	71/F	2012	MM and CML	Simultaneous	IgG-κ	MP, Bd, Ld	Imatinib	Chr		Offiah et al. [17]
16	64/F	2013	MM and CML	Simultaneous	IgA-κ	BD	Imatinib	Chr		Romanenko et al. [18]
17	62/F	2013	MM	17	IgG-κ	RT, VCD, VCDD, VRD	Dasatinib	Chr, PCR	P210	Ragupathi et al. [19]
18	77/M	2014	MM and CML	Simultaneous	IgG-κ	RT, BD	No treatment	Chr, PCR	P210	Maerki et al. [20]
19	60/M	2014	MM	48	IgG-κ	RT, Ld	HU, dasatinib	Chr, FISH		Alsidawi et al. [21]
20	63/F	2012	CML	120	IgG-κ	BD, Ld	Imatinib	Chr, PCR	P210	Pessach et al. [22]
21	68/M	2012	MM	54	IgG-*λ*	VAD	Imatinib	Chr, PCR	P210	Pessach et al. [22]
22	76/M	2015	CML	38	IgA-*λ*	No treatment	Imatinib	Chr, PCR		Ahn et al. [23]
23	51/F	2016	MM	Unknown	IgG	BD	Imatinib	Chr, PCR		Wolleschak and Heidel [24]
24	88/M	2016	MM and CML	Simultaneous	IgD-κ	VRD	Imatinib	Chr		Ali et al. [25]
25	76/M	2018	MM	28	IgG-κ	Ld	Dasatinib, bosutinib	Chr, PCR, FISH	P190	Our case

BJP, Bence Jones protein; MP, melphalan, prednisolone; RT, radiation therapy; HU, hydroxyurea; LOAD-IN, melphalan, ranimustine, vincristine, IFN-*α*; PSL, prednisolone; VCD, bortezomib, cyclophosphamide, dexamethasone; VCDD, VCD plus doxorubicin; VRD, bortezomib, lenalidomide, dexamethasone; Ld, lenalidomide, dexamethasone; VAD, vincristine, doxorubicin, dexamethasone; BD, bortezomib, dexamethasone; CBD, BD plus cyclophosphamide; Rd, lenalidomide, dexamethasone; TD, thalidomide, dexamethasone; Chr, chromosome; PCR, polymerase chain reaction; FISH, fluorescence in situ hybridization.
